# Quantifying Maternal Health Using Digital Phenotyping: Protocol for a Longitudinal Observational Study

**DOI:** 10.2196/77175

**Published:** 2025-10-08

**Authors:** Amanda Glime, Taysir Mahmoud, Soni Rusagara, Alysa St Charles, Devika Lekshmi, Ashley Peterson, Aarti Sathyanarayana

**Affiliations:** 1 Bouvé College of Health Sciences Northeastern University Boston, MA United States; 2 Khoury College of Computer Sciences Northeastern University Boston, MA United States; 3 Woman, Mother, and Baby Research Institute Tufts Medical Center Boston, MA United States; 4 Department of Obstetrics and Gynecology Tufts Medical Center Boston, MA United States

**Keywords:** digital phenotyping, longitudinal data, pregnancy, smartwatch, smartphone, ecological momentary assessment

## Abstract

**Background:**

We present a digital phenotyping protocol designed to continuously and objectively measure behavioral, physiological, and contextual data during pregnancy and the postpartum period using passive sensing from Garmin smartwatches and smartphones, along with active ecological momentary assessments (EMAs). This novel protocol uniquely adapts to the unpredictable timing of childbirth, spanning from the third trimester through 6 weeks post partum, to accurately capture critical temporal changes and maternal-infant outcomes. By providing high-frequency real-time data, this methodology offers comprehensive insights into pregnancy-related behaviors and physiological processes, overcoming the limitations of traditional retrospective self-report methods.

**Objective:**

We aim to develop a protocol for longitudinal data collection supporting digital phenotyping that is optimized for pregnancy and the postpartum period. This protocol leverages the pregnant population’s heightened interest in health and tracking. It aims to minimize the burden on the participants, increase retention, and assess the value of wearables compared to smartphones to determine the appropriate data collection methods.

**Methods:**

Data will be collected from 30 nulliparous participants from the start of the third trimester through 6 weeks post partum. This protocol uses 3 distinct 1-time surveys, alongside daily and weekly EMAs, to capture real-time maternal experience data. Passive maternal data—such as activity, vitals, sleep, and location—are collected via smartphones and Garmin smartwatches. Participants are expected to log data about the newborn after delivery through the mobile app Huckleberry. This protocol was developed in collaboration with the Northeastern University Sath Laboratory, which focuses on digital phenotyping and longitudinal data collection, and the Tufts Medical Center’s obstetrics and gynecology department, which has expertise in working with the pregnant population.

**Results:**

This study was funded in August 2024. Data collection is projected to run from October 2025 to July 2026. As of September 2025, the study has been approved, and recruitment and data collection are to begin. The results are expected to be published by August 2026. We plan to assess the retention rates, survey and EMA completion rates, wear time of the smartwatch without intervention, and data volume logged in the Huckleberry app. In addition, we will perform digital phenotyping to determine whether the data collected during pregnancy can be used to predict breastfeeding outcomes, delivery outcomes, and maternal-infant well-being.

**Conclusions:**

This protocol integrates the use of digital phenotyping in pregnancy and postpartum research, providing a novel method for capturing real-time indicators of maternal well-being. It will determine the expected rates of data completion and appropriate sample size using a power analysis for a more extensive future study. By integrating smartphone and wearable sensor data, this protocol has the potential to transform the way maternal health clinical interventions are designed and implemented in the future.

**International Registered Report Identifier (IRRID):**

PRR1-10.2196/77175

## Introduction

### Background

We present a digital phenotyping protocol specifically designed for pregnant populations using smartphones and Garmin smartwatches to continuously measure behavioral, physiological, and contextual data. Our design addresses the inherent challenges posed by pregnancy, including a nonfixed study duration and unpredictable delivery-triggered phase transition, by spanning from the third trimester through 6 weeks post partum. This protocol also takes into account the engagement-burden tradeoff with data collection and increased physiological variability during pregnancy through various data streams and development specifically for digital phenotyping, which is the in situ quantification of an individual’s phenotype using data from their digital devices, such as smartphones and wearable sensors [1]. This approach allows for the continuous objective measurement of behavior, physiology, and environmental context in someone’s everyday life, offering a detailed understanding at the individual level [1]. Data in this study are collected both passively via automatic sensing from ubiquitous devices and actively through ecological momentary assessments (EMAs), which are brief surveys completed by participants in their everyday environments. By overcoming the limitations associated with retrospective self-report methods, this approach allows for a more comprehensive understanding of pregnancy-related behaviors, physiological processes, and maternal and infant outcomes.

Conventional analytical techniques that rely on retrospective self-reporting can be affected by recall bias and thus fail to capture critical temporal changes [1]. In contrast, digital phenotyping uses high-frequency real-time data [1]. Previous perinatal digital phenotyping studies, such as the Mom2B study [2] and the Postpartum Mothers Mobile Study [3], have shown the feasibility of smartphone-based prediction of outcomes, EMA for post partum and breastfeeding, and mobile tracking of maternal health. Although the Mom2B study relied primarily on smartphone data, our protocol extends this work by integrating synchronized wearable data and multiple app-based platforms. We also adapted EMAs from the study by Demirci and Bogen [4] on primiparous women and the combination of EMA and smartphone data from the Postpartum Mothers Mobile Study [3]. By continuously and objectively capturing behavioral and physiological data, our protocol provides robust insights into the complex interactions influencing maternal and infant health outcomes. This comprehensive approach can significantly enhance our understanding of the pregnancy and postpartum experience. In addition, by collecting a multitude of data that adapts after participants deliver, we can understand participants’ behavior in a more comprehensive way than digital phenotyping studies that stay the same during different stages of pregnancy or the postpartum period.

### Nonfixed Study Duration

The first challenge that this protocol must address is the nonfixed study duration.

The duration of an individual’s pregnancy is highly variable, and in clinical practice, the estimated due date (EDD) is 40 weeks of gestational age [5]. In the Early Pregnancy Study [6], even after excluding pregnancies affected by complications or preterm births, the variation in human gestational length was 37 days. Only approximately 5% of people deliver on their assigned 40-week gestational age EDD, and 66% deliver within 7 days of their EDD [5]. The variability in delivery timing means that although the duration of the third trimester is defined as 12 weeks, beginning at 28 weeks of gestation and culminating at 40 weeks of gestation [7], many people will have a third trimester that is shorter or longer. The duration of the third trimester can range from less than 1 week, if the baby is delivered at 29 weeks, to approximately 14 weeks. For example, a participant delivering at 30 weeks 0 days gestation would have 2 weeks of data collected in the third trimester and 6 weeks of data collected in the postpartum period for a total of 8 weeks. Conversely, a participant delivering at 41 weeks and 6 days of gestation would have 14 weeks of data collected in the third trimester and 6 weeks of data collected in the postpartum period for a total of 20 weeks. The nonfixed third-trimester study duration creates a challenge, as compensation in a study is typically for a set amount of time. In addition, data storage amounts cannot be pre-estimated, and research apps typically need to know the study duration before commencement. In addition, to best understand pregnancy, EMA questions need to transition from prenatal-focused questions to postpartum-focused questions. Given these inherent biometric challenges, we developed a protocol that adapts to both longer and shorter study durations.

### Unpredictable Delivery-Triggered Phase Transition

The variance in the third-trimester duration for participants creates the challenge of an unpredictable delivery-triggered phase transition, which is the change in EMA questions that occurs because of the participant delivering their baby. We collect data in 2 phases, antepartum third trimester and postpartum period, which are segmented by delivery. There is no way to know for certain exactly when the baby will be delivered, and the delivery date will be different for each participant. There will be a discrepancy between the EDD (40 weeks) and the actual delivery date, and between the actual delivery date and the date on which the research team becomes aware that the participant’s pregnancy has ended. This may lead to a delay when the postpartum EMA questions are triggered. The unpredictable delivery-triggered phase transition will also lead to some participants providing a different amount of data before and after delivery, with participants who have a longer third trimester providing a greater amount of data before delivery. This variability introduces complexity when developing a longitudinal data collection protocol aimed at capturing critical behavioral and physiological changes during pregnancy, the puerperium, and the postpartum condition. The protocol must account for transitioning from prenatal data collection streams to the postpartum data collection streams in a way that can be implemented variably after delivery without leading to delays in postpartum data collection.

### Engagement-Burden Tradeoff

This protocol aims to investigate the engagement-burden tradeoff. Research data suggest that pregnancy is a health care state associated with high rates of engagement with online searches. One study reported that 90% of pregnant individuals obtained supplemental health care information online [8]. In addition, 50% of pregnant individuals installed a health-tracking app on their phones during pregnancy [9]. This provides a unique opportunity to leverage this increased engagement to collect more data than is typically collected from other populations. However, we must balance this opportunity with minimizing the burden on the participant while still emphasizing increased engagement. Previous studies have prioritized minimizing the burden on participants by including only 1 detection device, collecting fewer data streams, or implementing either passive or active data collection, and not both simultaneously. This protocol must leverage the opportunity for increased data collection while also incorporating multiple touchpoints with clinicians and the use of apps and devices that participants are familiar with.

### Vulnerable Population

The pregnant population is vulnerable and experiences increased physiological, psychological, and social variability [10,11]. Several physiological and potentially pathophysiological changes occur during pregnancy [12,13]. Subjective data regarding these changes are collected with surveys at multiple points throughout the pregnancy and after delivery. To address the vulnerability of pregnant participants, this protocol must have questions tailored to and validated for the pregnant population and not make assumptions about the participants. Objective physiological data are obtained via passive sensing with a wearable device.

Many previous digital phenotyping studies investigating pregnancy have focused exclusively on the postpartum or perinatal period or have used the same protocol for both phases [2-4]. By focusing exclusively on 1 period, key changes that occur between the pregnancy and postpartum periods are missed. In addition, these protocols did not need to account for the uncertainty in delivery timing or adjust their protocol during the postpartum period due to having 1 consistent protocol or focusing on 1 period [2-4]. In contrast, our protocol uniquely spans the duration of each participant’s third trimester and resumes immediately during the postpartum period for 6 weeks of new data collection. Our protocol supports digital phenotyping by combining passive data collection from Garmin smartwatches and participant smartphones with active labeling through EMAs. Over an approximate 18-week period, from the third trimester through the 6-week postpartum period, we continuously track behaviors and physiological data relevant to pregnancy outcomes, such as delivery, newborn feeding practices, and mental health and well-being.

## Methods

### Study Design

**Figure 1:**
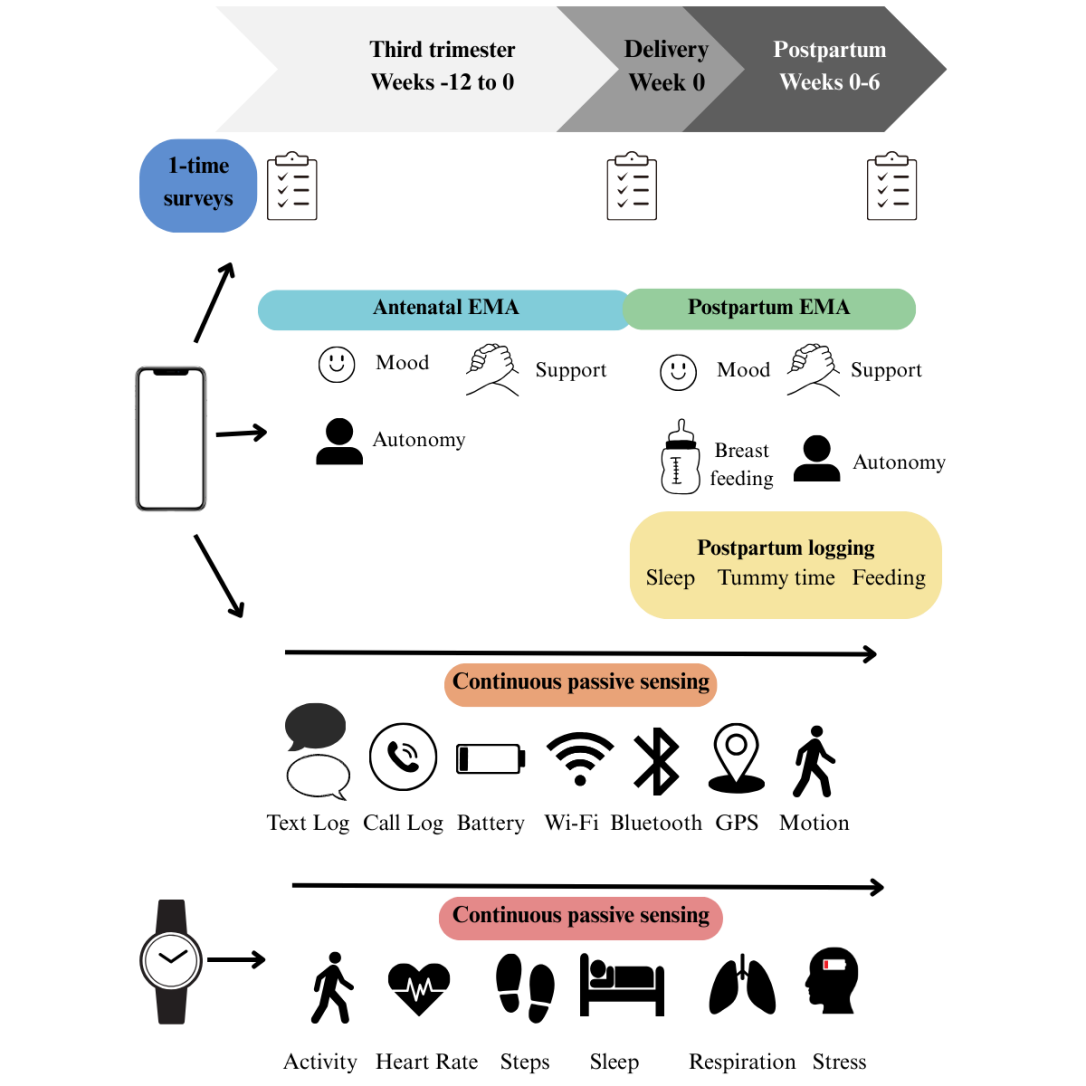
Overview of the study procedure and timeline. EMA: ecological momentary assessment.

This protocol collects data passively through a smartphone (Beiwe app [14]) and smartwatch (Labfront application [15]). It collects data prospectively and actively through participant survey completion, EMAs, and participant data logging in the Huckleberry app [16]. The use of the Beiwe app addresses the nonfixed study duration for EMAs and smartphone data by allowing flexibility in study length. The use of the Labfront application for smartwatch collection also allows for flexibility in the length of the study. The unpredictable delivery-triggered phase transition is also managed by the Beiwe app, as the platform allows us to trigger a change in EMAs after delivery. The engagement-burden tradeoff is addressed by adding more active data collection through EMAs and having participants log data in the Huckleberry app. We allow participants to log their data into the Huckleberry app whenever it is convenient for them and balance the burden of EMAs by implementing this only once a day. Finally, to ensure that the protocol is pertinent for this population with increased physiological variability, we have reviewed and adapted all 350 survey questions and 14 unique EMA questions. In addition, we incorporate all study visits with in-person medical appointments and have clinicians and doulas on staff to help with any situation that may arise (Figure 1).

### Participants

An initial cohort of approximately 30 nulliparous (females who have never given birth to a living child) participants will have data collected from the start of their third trimester through the 6-week postpartum period. A starting point of 30 participants was selected to ensure fair compensation. The inclusion criteria are that the participants should (1) be pregnant, (2) be between 14 and 28 weeks of gestation, (3) be nulliparous, (4) be aged ≥18 years, (5) own a smartphone, and (6) speak English. The participants will be enrolled before 28 weeks of gestation. Participants must own a smartphone to be able to answer the daily surveys and collect data. Participants must speak and read English, given that the daily survey questions are currently provided in English only. Participants will be excluded if they (1) are unable to provide consent, (2) have a pre-existing diagnosis of anxiety or depression, (3) are unable to wear a smartwatch, or (4) have a medical contraindication to breastfeeding. Participants who are unable to provide consent will be excluded. We will exclude participants with a formal diagnosis of anxiety or depression to reduce the effect of confounding variables, as pre-existing anxiety or depression can impact pregnancy outcomes [17]. Participants must also be able to breastfeed, as we will track breastfeeding rates and attitudes during the postpartum period as one of our main outcomes. Finally, someone who is unable to wear a smartwatch for the entire period is excluded, as they would not be able to collect physiological data such as heart rate, sleep, and activity.

### Recruitment

Participants will be recruited in 4 ways to allow for maximum reach. This population has increased contact with physicians, allowing for increased opportunities for recruitment. First, informational posters will be affixed to walls in the waiting room at the Tufts Medical Center’s obstetric clinical areas. Second, the Tufts Medical Center’s obstetrics and gynecology prenatal practice patients will be messaged with a notification regarding the study at around 20 weeks of gestation through a health care provider-patient online portal. Third, participants can be recruited through phone calls from 20 to 24 weeks of gestation. Finally, participants can be approached at a midtrimester (14-28 weeks of gestation) appointment in person. Eligible participants will be identified by the Tufts Medical Center research team using information from their medical charts. This screening log will be stored in a password-protected folder and deleted after the trial completion. To reduce the time spent in the obstetrics and gynecology office and for patient convenience, participants will be able to walk through the informed consent process over the phone if they elect and sign consent forms electronically. Once individuals choose to participate, they will be guided by a research coordinator regarding the enrollment process, which starts with scanning a QR code that brings the users to a brief enrollment survey. This will be conducted at an in-person prenatal care appointment (at approximately 24-28 weeks of gestation). This enrollment survey provides the participants’ email and due dates to the Northeastern University research team, allowing the instant transfer of information from people who are only interacting in person at the Tufts Medical Center with the Tufts staff to the Northeastern University research team. Participants will install all the necessary applications and set up their smartwatch with guidance from the research coordinator and a study-specific installation physically provided to them.

### Data Collection

#### Overview

Data will be collected in the wild over approximately 18 weeks using five methods: (1) surveys (1-time baseline assessments at key perinatal milestones), (2) passive data collection from smartphones, (3) passive data collection from Garmin smartwatches, (4) EMAs, and (5) postpartum logging via the smartphone app.

#### Surveys

##### Overview

Three questionnaire surveys will be completed by participants throughout the study. These surveys are used to obtain information at each milestone (start of the third trimester, delivery, and 6 weeks post partum) about the mother’s experiences and well-being.

##### Survey Technology

The surveys will be delivered as an email link using the Qualtrics platform and can be completed online via a smartphone or computer.

##### Survey Metrics

The surveys consist of questions from the following validated questionnaires: National Institute of Health demographics form [18], Mother Infant Lactation Questionnaire [19-21], Pregnancy Risk Assessment Monitoring Systems [22,23], Hunger Vital Sign [24-27], Housing Instability Index [28,29], General Health Questionnaire [30,31], Big 5 Inventory-10 [32,33], Body Understanding Measure for Pregnancy Scale [34], Maternal Health Literacy Inventory in Pregnancy [35,36], Eating Attitudes Test [37-39], Infant Feeding Intention Scale [40,41], Abuse Assessment Screen [42,43], Childbirth Experience Questionnaire [44-46], Iowa Infant Feeding Attitudes Scale [47,48], Edinburgh Postnatal Depression Scale [49-54], Perinatal Posttraumatic Stress Disorder Questionnaire II [55,56], Neonatal Eating Assessment Tool [57], Breastfeeding Self-Efficacy Scale [58,59], and the Self-Acceptance Scales for Pregnant Women [60,61]. The final survey will also include questions regarding their overall experience within the study.

##### Survey Scheduling

The first of the 3 surveys will be sent to the participants at 32 weeks of gestation. The second survey will be sent to each participant when they self-report the date of delivery to the research team. The third and final survey will be sent to the participants at 6 weeks post partum.

For all 3 surveys, participants will receive follow-up emails containing reminders about the surveys every 3 days until the survey is completed. If the participants do not self-report delivery to the research team, and it is 1 week or more past their due date, the research team will message the participant. The Tufts research team will also have access to patients’ records to confirm the pregnancy status.

#### Passive Smartphone Data

Passive smartphone data are collected to obtain real-time objective data about participants’ behavior and activity.

##### Passive Smartphone Technology

Passive data will be collected by a smartphone. Participants will use their own smartphones, and data will be collected through the Beiwe app, which has been used in other studies to collect data on the pregnant population [2,14].

##### Passive Smartphone Data Metrics

The data collected by the Beiwe app are shown in Table 1.

**Table 1:** Smartphone data variables.

Sensor	Description	Frequency	Compatible device
GPS	Location (latitude and longitude)	Every 10 min	iOS and Android
Accelerometer	Physical activity (this can be sitting, standing, walking, in a vehicle, running, or on a bicycle)	Continuous	iOS and Android
Gyroscope	Orientation or angular velocity (rotation or positioning of the phone)	Continuous	iOS and Android
Magnetometer	Magnetic fields (compass)	Continuous	iOS
Time	Date and time	Continuous	iOS and Android
Wi-Fi	Wi-Fi connection status	Every 15 min	Android
Bluetooth	Bluetooth scan (search for name and devices nearby)	Every 15 min	Android
Reachability	Network status	In response to when the network status changes occur	iOS
Battery status	Battery percentage level and charging status	Continuous	iOS and Android
Call and SMS text message logs	Dump of daily call log and SMS text message log	Once daily	Android

##### Passive Smartphone Data Scheduling

Smartphone data are recorded continuously for the entirety of the study. Data collection is only paused when the phone is shut down.

#### Passive Smartwatch Data

Smartwatch data are collected passively to obtain real-time objective data about the participants’ behavior and activity whenever the participant is wearing the watch.

##### Passive Smartwatch Technology

Participants will be provided with a Garmin Vivosmart smartwatch that synchronizes with the Labfront Companion application to collect passive data. The Garmin Vivosmart was selected due to its ability to track interbeat intervals and previous history of safe use in this population [62,63], along with being recommended by Labfront. In addition, Garmin Vivosmart watches have been widely used in research and have been shown to collect accurate data [64-66].

##### Passive Smartwatch Data Metrics

The data collected by Garmin Vivosmart are shown in Table 2. The sampling rates can be adjusted and will fall within the ranges provided.

**Table 2:** Smartwatch data variables.

Variable	Description	Sampling rate
Daily summary	Summary of the data collected from each day	Daily
Activity summary	Summary of activity types (eg, walking, running, and cycling) from each day and their intensities	15 min
Actigraphy	Monitors rest and activity cycles using an accelerometer	10 s to 1 min
Sleep	Sleep duration and stages are calculated using heart rate and actigraphy data	Auto-detect
Heart rate	Beats per min measured using photoplethysmogram sensors	1 s to 60 min
Stress	Numerical output depicting stress level calculated based on heart rate and activity	10 s to 60 min
Steps	Number of steps calculated using an accelerometer	1 min to 15 min
Respiration	Breathing rate measured in breaths per min	10 s to 60 min
Interbeat interval	Time interval between different beats of the heart	Each heartbeat

##### Passive Smartwatch Data Scheduling

Smartwatch data are recorded continuously for the entirety of the study. Data collection is paused only when the watch is shut down, dies, or is removed.

#### EMA Data

##### Overview

EMAs are used to provide additional insights into passive data by collecting real-time insights into the factors that influence breastfeeding outcomes. EMAs have been widely used in psychological and health research to assess momentary states and situational factors that influence outcomes over time [67]. Using EMAs alongside traditional longitudinal methods, this protocol offers a novel approach in capturing breastfeeding behaviors and maternal health with real-time accuracy.

##### EMA Technology

EMAs will be delivered via a smartphone through the Beiwe app [14].

##### EMA Metrics

The daily EMA survey includes questions adapted from the Perceived Stress Scale [68-70] to map responses to the circumplex model of affect [71]. These questions pertain to mood, confidence, and personal autonomy. Weekly EMA surveys include EMA-adapted questions from the Perceived Stress Scale [68-70], Self-Acceptance Scale for Pregnant Women [60,61], Maternal Antenatal Attachment Scale [72,73], and Breastfeeding Effectiveness Scale [74-76]. These questions have been adapted to follow a 5-point Likert Scale. After delivery, the EMA survey will include additional daily questions focused on breastfeeding attitudes and complications during the postpartum period [4,77].

##### EMA Scheduling

The EMA questions are delivered daily. Once a week, additional weekly questions are added to the daily survey. The EMAs are delivered each day at 6 PM, as this is the time of day when participants are most likely to answer [78,79]. At the end of the day, the participants are likely to have completed their daily activities and can reflect on their experiences more thoroughly. This timing has also been used in previous EMA studies to promote thoughtful responses and improve participant engagement [3,49,80].

To enhance participant retention and commitment throughout this longitudinal study, we designed each EMA to be brief and minimally intrusive, with completion times of approximately 2 minutes. This is shorter than most previous EMA studies, such as the study by Burke et al [81], in which participants completed 3 EMAs per day, each lasting 3 to 5 minutes. Given the extended nature of this protocol study, this optimized approach is expected to reduce participant burden and enhance the reliability of longitudinal data collection.

**Figure 2:**
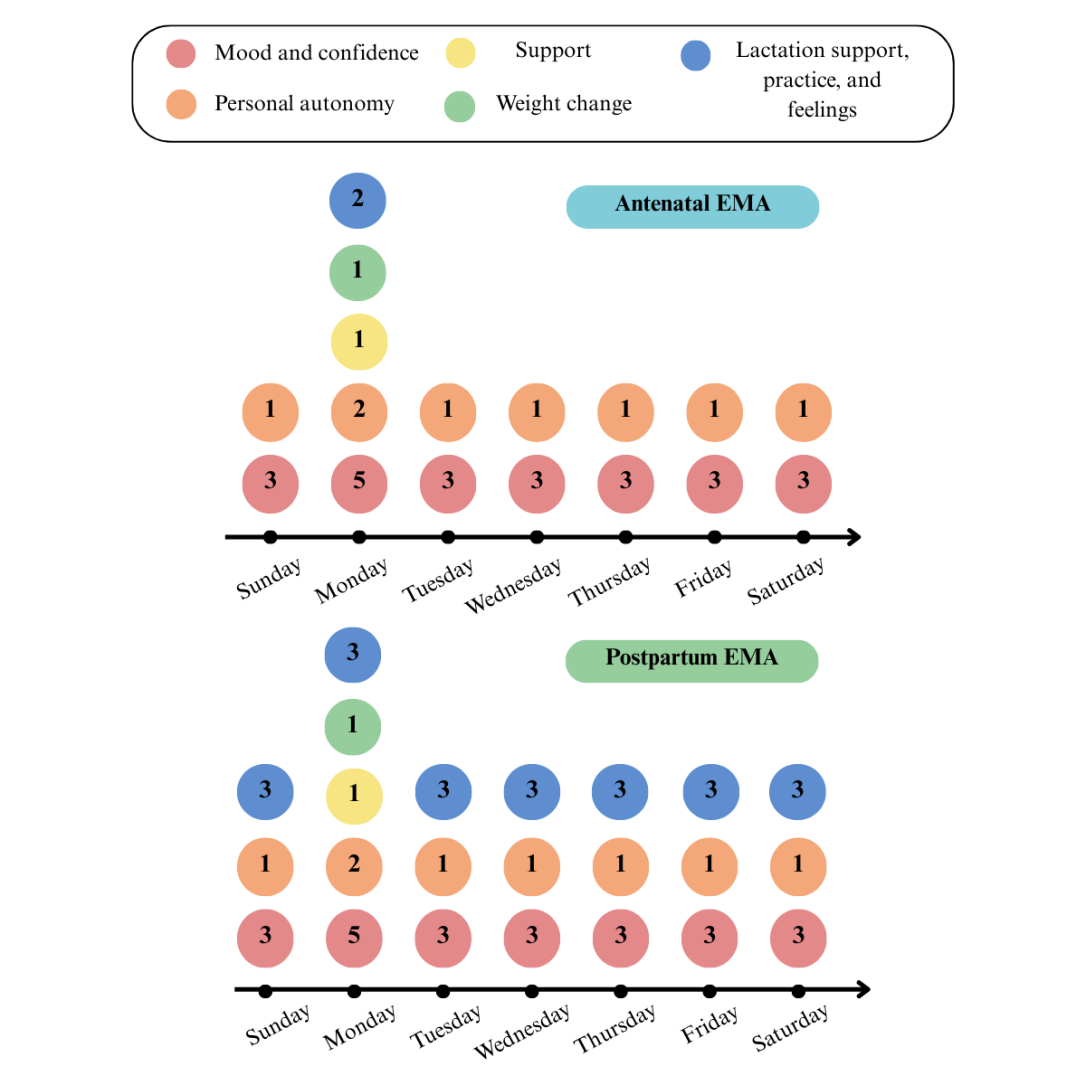
Ecological momentary assessment (EMA) schedule showing the number of questions asked daily during pregnancy and the postpartum period, with the number in each circle depicting the number of questions asked.

The additional weekly EMA questions are delivered on Mondays at 6 PM to increase compliance, as EMA surveys typically receive higher compliance earlier in the week, on weekdays, and in the evening [78,79] (Figure 2).

#### Postpartum Logging Data

The data logged postpartum is about the infant but is logged manually by the mothers.

##### Postpartum Logging Technology

Participants will log data about their babies using the smartphone app Huckleberry [16].

##### Postpartum Logging Metrics

The metrics collected for this study through the Huckleberry app are feeding data, tummy time, and sleep data [16]. For feeding, mothers will log when the babies were fed, how long they were fed, what they were fed, and the amount they were fed. For tummy time, mothers will record when the babies did the tummy time and its duration. For sleep, participants will log when their babies sleep and for how long.

##### Postpartum Logging Scheduling

Participants will log the information regarding their newborn’s sleep, feeding, and tummy time. They will also be able to do this after the event has occurred. They will record the information from delivery to 6 weeks post partum. At the conclusion of the study, participants will download the data from the Huckleberry app and share it with the research team. The participants will be able to upload the link provided by the Huckleberry app into a Qualtrics survey, which eliminates the need for users to go through the process of downloading large files and uploading them again, and keeps the data secure, as Qualtrics is a Health Insurance Portability and Accountability Act–compliant platform. This will enable the easy transfer of data from the participants to the research team.

### Data Synchronization and Storage

To ensure consistency across the Beiwe and Huckleberry apps and the Labfront application, all data will be aligned using standardized timestamps. Before study initiation, each participant’s smartwatch will be synchronized to the designated study time zone. During data processing, all streams will be harmonized based on these timestamps, allowing for the precise alignment of physiological, behavioral, and EMA data. A unique study ID, whose key is stored separately from the data, will link participant data across platforms, ensuring the proper integration of multiple data sources. This approach not only enables reliable data merging but also enhances robustness by allowing the research team to account for missing information in 1 platform with corresponding data from another. All data are stored in password-protected folders on the Northeastern University server. Only approved members of the research team will have access to these data. Data will be deidentified as much as possible.

### Data Checks and Collaboration

Data quality checks will be performed every morning between 8 AM and 10 AM by the research team to assess completion rates and wear time and to ensure that data values are within the expected range. Low wear time, missing data, and missing surveys will result in an email being sent from the research team to the participants to remind them of how to properly wear the watch, open the Garmin application to synchronize their daily watch data, and answer the surveys. If missing data are due to a technical difficulty, the research team will find a solution or give the participant a new device. If participants no longer meet the requirements, for example, can no longer wear the watch or are leaving the country, they will be removed from the study. If participants withdraw or are removed from the study, their data will be deleted and not included in the analysis. The Northeastern University and Tufts Medical research teams will have regular meetings to ensure proper trial conduct and to communicate any changes that need to be made. The research team will also, nonsystemically, keep track of any reported harm that occurs to participants; however, we predict that harm will be unlikely to occur. Potential risks for participants include discomfort or rash from the smartwatch, emotional discomfort when answering EMA questions or survey questions, and a potential loss of confidentiality and data security.

### Ethical Considerations

This protocol has been approved by the Tufts Health Sciences Institutional Review Board (STUDY00005835 protocol version 5.14.2025). This study is being conducted in collaboration with the Tufts Medical Center, which is Health Insurance Portability and Accountability Act–compliant. All members of the research team have undergone training on research involving human participants. We will obtain informed consent from all participants. The privacy and confidentiality of participants will be protected. Participants are compensated for their participation in this study.

## Results

The pilot study following this protocol is expected to begin in October 2025. The primary goal of this pilot study is to evaluate the feasibility of our protocol for implementation in future larger-scale studies. The quality of the watch and smartphone data will be assessed daily, evaluating the scalability and adaptability of the methods for use with another cohort. This daily check includes running a script to analyze the wear time and completion rates and verifying that the data values are within the normal range. Participant engagement and retention rates will be assessed through metrics, including survey and EMA completion rates, self-logging data frequency, timeline of survey completion, perceived participant burden, usability of smartphone apps and smartwatch applications, and overall participant experience. We will also measure the data volume logged by participants in the Huckleberry app to understand the expected amount of data to be logged by future participants. We will also determine the amount of data that the smartwatch adds to the study and how much the data impact digital phenotyping. This will help to determine the advantage of including the watch in addition to the smartphone. Finally, digital phenotyping, starting with mood, activity, stress, and sleep, will be conducted to determine whether we can use the data collected during the third trimester to determine delivery outcomes, baby behavior, breastfeeding outcomes, and postpartum depression. These results will provide evidence on the viability of digital phenotyping using longitudinal pregnancy data, supporting a comprehensive investigation into third-trimester predictors of pregnancy outcomes.

## Discussion

### Anticipated Findings

#### Overview

Pregnancy is a period of continuous and significant physiological, psychological, and social change, creating an opportunity for digital phenotyping to passively capture this period. The variability in due date causes a nonfixed study duration and unpredictable delivery-triggered phase transition. Pregnant participants also have a heightened interest in their health, creating an opportunity to leverage their increased engagement to collect more data, but requiring consideration of the participant burden. Finally, pregnant women are increasingly vulnerable due to physiological changes. We have addressed these challenges in the subsequent sections.

#### Adapting to Nonfixed Study Duration

The variability in pregnancy duration introduces a fundamental challenge for study design, as the length of participation cannot be standardized across all individuals. This unpredictability affects compensation plans, technical planning, and survey logic within the research app. To address this, our protocol was designed to be flexible, allowing for individualized adaptation to both shorter and longer durations of data collection. Most apps require a set period for the study duration. We did not use an in-house application; therefore, we had to negotiate a contract such that the timeline is not fixed. Future apps in the research space should plan for this, as pregnancy studies will always have variation in duration. In addition, at the start of the study, data storage should be planned for the full duration of the pregnancy. As the study proceeds, it may be possible to predict preterm or postmature births to better optimize data storage. For compensation, we set a limit and communicated this to the participants. Other forms of motivation for participation should also be considered to supplement the financial component. One potential alternative form of compensation is to share weekly updates of the participants’ trajectories.

#### Implementing Delivery-Triggered Phase Transition

Another key challenge in developing this protocol is the unpredictable timing of the transition from prenatal to postpartum data collection. Because delivery dates may not be immediately known to the research team, it is difficult to ensure timely updates to survey content. This requires a flexible protocol capable of managing delayed notifications and uneven data collection across the study phases. This was a significant challenge that required additional consideration. We used an application that is optimized for protocols with an intervention at a fixed date. The system is not set up for variation in the period before the intervention, which in our case is childbirth. To mitigate this challenge, we ask participants to self-report their delivery directly to the research team; however, this may cause a delay in notifying the research team. Once reported, the research team can manually trigger a change from antenatal to postpartum EMAs. This process is streamlined so that within 24 hours of the participant notifying their delivery team, the research team can trigger the phase transition. We have designed a failsafe method to reduce potential large delays—the research coordinator at the clinical site will review due dates and verify if a participant has delivered on a weekly basis. In addition, if a participant misses this report by a few days, we will still have their passively recorded data and any EMA questions that are asked in both periods.

#### Minimizing Participant Burden

This protocol seeks to strategically optimize a larger amount of data collection by leveraging high engagement during pregnancy and minimizing the participant burden. While pregnancy presents a unique opportunity for increased data collection, studies must remain unobtrusive and align with existing tools and routines to maintain sustained participation. To leverage the increased engagement of the pregnant population, we use multiple apps to collect data from various streams, including allowing users to input data about their babies’ behavior in addition to their own data. We use the Huckleberry app for the manual input of data, as Huckleberry reports that the app is already used by more than 4 million parents worldwide [82]. Furthermore, we use both smartphones and smartwatches to collect continuous data. We also streamlined the EMA delivery by minimizing the number of questions asked and asking the questions only once a day. By delivering the EMAs at 6 PM, we reach participants during the time they are most likely to answer while considering typical bedtimes during pregnancy. We also ask a series of questions, requiring only a few minutes each night to reduce the time required of each participant.

#### Protecting Participant Vulnerability

Pregnancy is a vulnerable time with complex physiological, psychological, and social changes, requiring sensitive and tailored data collection approaches. This protocol is streamlined according to the actual clinical care that participants receive during pregnancy and the postpartum period. Participants are recruited remotely and in person at already-scheduled appointments throughout the second trimester. Enrollment, including providing devices and meeting with the research coordinator, also occurs at an already-scheduled appointment. After the study, the devices are returned to the clinic when participants come in for their next postpartum appointment. In addition, we optimized the phrasing of the questions to be sensitive to the vulnerability of the pregnant population and used questionnaires developed specifically for pregnancy. For example, instead of using generic depression questionnaires, such as the Patient Health Questionnaire-9, we use the Edinburgh Postnatal Depression Scale, which is tailored to postpartum participants.

### Limitations

Certain aspects of this protocol require manual and timely tasks to be done. An example of this is that available data collection apps supporting digital phenotyping are not set up to account for a nonfixed study length. The exact data of childbirth is unpredictable, and a delay from self-reporting and implementing the change in the EMAs should be anticipated. Handling this transition phase manually is increasingly tedious as the study becomes larger, implying that scalability is constrained by the time of the research coordinator. Another example of a manual task is transferring the data collected in the Huckleberry app to the research team. In the future, an app that provides the participant with their health information should also provide research protocols for a more seamless experience. An additional limitation of the protocol is that it requires participants to wear the wearable device provided by the study team. It would be better for participants if the data collection tools were device-agnostic, allowing them to use their own devices. Finally, this protocol may have a higher than usual dropout rate because of not meeting the criteria of a viable pregnancy, due to the many complications that can occur during pregnancy. We mitigate this partially by starting data collection as late as the third trimester, which reduces the opportunity for complications compared to data collection starting earlier in the pregnancy.

### Future Work

We intend to use participant feedback and completion rates from this study to refine the protocol for a future large-scale study of pregnancy with varying conditions. We will also use the results of longitudinal data analysis from this study to determine the effect size of factors, such as activity, feelings of support, mental health, mood, and breastfeeding intentions during the third trimester, on pregnancy, breastfeeding, and mental health outcomes. These estimated effect sizes will inform power analyses in future research endeavors.

## Data Availability

The datasets generated during this study are available from the corresponding author upon reasonable request.

## Abbreviations

EDD: estimated due date
EMA: ecological momentary assessment
